# Dysfunctional Metacognitive Beliefs Are Associated with Decreased Executive Control

**DOI:** 10.3389/fpsyg.2017.00593

**Published:** 2017-04-19

**Authors:** Brage Kraft, Rune Jonassen, Tore C. Stiles, Nils. I. Landrø

**Affiliations:** Clinical Neuroscience Research Group, Department of Psychology, University of OsloOslo, Norway

**Keywords:** metacognitions, metacognitive beliefs, executive control, executive functions, rumination, worry, depression

## Abstract

Dysfunctional metacognitive beliefs (“metacognitions”) and executive control are important factors in mental disorders such as depression and anxiety, but the relationship between these concepts has not been studied systematically. We examined whether there is an association between metacognitions and executive control and hypothesized that decreased executive control statistically predicts increased levels of metacognitions. Two hundred and ninety-nine individuals recruited from the general population and outpatient psychiatric clinics completed the Metacognitions Questionnaire-30 and three subtests from the Cambridge Neuropsychological Test Automated Battery corresponding to the three-component model of executive functions. Controlling for current depression and anxiety symptoms, decreased ability to shift between mental sets was associated with increased negative beliefs about the uncontrollability and danger of worry and beliefs about the need to control thoughts. The results suggest a basic association between metacognitions and executive control. Individual differences in executive control could prove important in the personalization of metacognitive therapy.

## Introduction

Metacognition refers to the control, modification, and interpretation of thoughts (Wells and Cartwright-Hatton, 2004) and is conceptually closely related to executive control (Fernandez-Duque et al., 2000). Executive functions control and coordinate low-level cognitive processes to guide behavior toward a goal (Banich, 2009). Both executive control mechanisms and metacognition have been highlighted in recent models of mental disorders such as major depression and generalized anxiety disorder (Wells, 1995; Matthews and Wells, 2004; Eysenck et al., 2007; Joormann et al., 2007). However, whether metacognitive beliefs and executive control are empirically related has not been studied systematically.

Impaired executive control has been consistently linked to depression (Snyder, 2013) and rumination (Yang et al., 2016). Rumination is a repetitive and passive focus on one's depressive symptoms and is an important factor in depression (Nolen-Hoeksema et al., 2008). Having certain dysfunctional metacognitive beliefs (“metacognitions”) predicts rumination and depression (Papageorgiou and Wells, 2009). Common metacognitions in depression include beliefs that rumination will help find answers about the causes of one's depression and that depressive thoughts are uncontrollable and damaging (Papageorgiou and Wells, 2001). Such metacognitions lock the individual in a perseverative cycle of negative thinking (Matthews and Wells, 2004). Likewise, an inability to disengage attention from negative thoughts has been proposed as a central neurocognitive mechanism in the consolidation of depressive symptoms (Koster et al., 2011).

Individuals with a history of depression tend to worry about relapsing (Wells and Carter, 2002; Spada et al., 2008b; Sarisoy et al., 2014). Rumination and worry may be conceived as similar cognitive processes described in different research contexts (Watkins et al., 2005) and can be conceptualized collectively as persistent negative thinking (Beckwé et al., 2014). Metacognitions about worry have also been proposed as a promising vulnerability marker of depressive relapse (Halvorsen et al., 2015).

The self-regulatory executive function (S-REF) model of emotional disorders integrates information processing research with Beck's schema theory (Wells and Matthews, 1996). Central to this model is how metacognitions reinforce inflexible and maladaptive coping responses. Metacognitions provide top-down generic procedures for coping (“rumination is helpful”). Input from low-level networks (negative thought intrusions) activate a coping response (rumination), and an online process termed the supervisory executive controls and evaluates the effectiveness of the coping response. The supervisory executive appears to overlap with the concept of executive control, and Wells and Matthews (1996) has proposed attentional capacity and reduced cognitive flexibility as important factors in the modification of metacognitions.

The executive functions can be conceived as three distinct but related components: “shifting” (between tasks or mental sets), “updating” (and monitoring of working memory representations) and “inhibition” (of prepotent responses; Miyake et al., 2000). Depression is associated with reduced performance in all three components (Snyder, 2013); a recent meta-analysis indicated that reduced shifting and inhibition (but not updating) contributes to a ruminative thinking style (Yang et al., 2016).

Very little is known about the neurocognitive correlates of metacognitions. Spada et al. (2010) found that metacognitions about worry were related to self-report of decreased ability to shift and focus attention. However, it is important to note that self-reports of executive control and cognitive test performance have weak or no correlation, and self-reports of executive control are actually better explained by emotional symptom load (Løvstad et al., 2016). Using an emotional Stroop task, Kaur et al. (2011) found a positive correlation between metacognitions and attentional bias for health words after health anxiety induction. However, this study only provided attention bias scores, and no measures of executive functions corresponding to the three-component model by Miyake et al. (2000). Thus, whether metacognitions are related to executive control remains unclear.

The main aim of the present study is to examine whether there is a basic association between metacognitions and executive control as measured by objective, well-standardized neuropsychological tests. We hypothesize that: (1) metacognitions and executive control are associated, and (2) decreased executive control statistically predicts increased levels of metacognitions after controlling for current depression and anxiety symptoms.

## Methods

### Participants

Participants were recruited by newspaper advertisements and posters, and from outpatient psychiatric clinics in Norway. They received oral and written information about the main aim of the study, which was to examine cognitive control and brain function in subjects with major depression and in healthy subjects. The inclusion criteria were age 18–65 years and fluency in Norwegian. All participants provided written informed consent in accordance with the Declaration of Helsinki, and the study was carried out in accordance with the recommendations of the Regional Committee for Medical and Health Research Ethics in Norway and the Norwegian Social Science Data Services.

### Measures and procedure

Trained psychologists performed evaluations for mental disorders using the Structured Clinical Interviews for DSM-IV criteria I and II (First and Gibbon, 2004). We excluded participants with a history of neurological disorders, bipolar disorder, or psychosis. Education level was classified according to the International Standard Classification of Education (ISCED; UNESCO, 1997). Participants completed Beck's Depression Inventory II (BDI; Beck et al., 1996) and Beck's Anxiety Inventory (BAI; Beck et al., 1988) to measure current symptoms of depression and anxiety, respectively. General cognitive function was examined using Picture Completion (PC) and Similarities (SI) subtests from the Wechsler Adult Intelligence Scale Third Edition (WAIS-III; Wechsler, 1997).

### Metacognitions

To measure metacognitions, participants completed the Metacognitions Questionnaire-30 (MCQ-30; Wells and Cartwright-Hatton, 2004). The MCQ-30 is a 30-item questionnaire where the respondent is asked to rate how much they agree with a specific statement on a 4-point Likert response scale: 1 (*do not agree*), 2 (*agree slightly*), 3 (*agree moderately*), and 4 (*agree very much*). The items generate five subscales: positive beliefs about worry (MCQ-PBW), which measures the extent to which a person believes that perseverative thinking is useful; negative beliefs about the uncontrollability and danger of worry (MCQ-NBW), which assesses the extent to which a person thinks that perseverative thinking is uncontrollable and dangerous; cognitive confidence (MCQ-CC), which assesses confidence in attention and memory; beliefs about the need to control thoughts (MCQ-NCT), which assesses the extent to which a person believes that certain thoughts should be suppressed; and cognitive self-consciousness (MCQ-CSC), which measures the tendency to monitor one's own thoughts and focus attention inwards. Internal consistency for the MCQ-30 is good (Cronbach's alphas from 0.72 to 0.93) and test–retest reliability is acceptable (*r* from 0.59 to 0.87; Wells and Cartwright-Hatton, 2004; Spada et al., 2008a).

### Neuropsychological assessment of executive control

Executive control was assessed using three subtests from the Cambridge Neuropsychological Test Automated Battery (CANTAB, 2009). Subtests corresponded to the three-component model by Miyake et al. (2000): the Intra-Extra Dimensional Task (ID/ED) measures shifting, the Spatial Working Memory Task (SWM) measures updating, and the Stop-Signal Task (SST) measures inhibition. The ID/ED requires participants to pay attention to different examples within a stimulus dimension and shift attention from one set of stimuli to a new, formerly unimportant set of stimuli across nine stages. Shifting ability is operationalized as total errors adjusted for whether the entire task is completed. A high total error adjusted score represents reduced shifting ability (Kaplan et al., 2006). The SWM requires participants to search through several boxes to locate tokens. After a token is located, it will not reappear in the same box during that same trial. Accuracy of working memory is operationalized as the between-trial errors score, which is calculated when the subject searches for a token in a box where a token had been found in a previous trial. High between-trial error scores represent working memory failures (Owen et al., 1990). The SST requires the subject to override a prepotent go response when presented with an infrequent stop signal (a beep). Inhibitory efficiency is operationalized as a stop-signal reaction time, which is estimated through automatic adjustment of the delay between the go stimulus and the stop signal. A higher stop-signal reaction time represents reduced inhibition ability (Logan et al., 1997).

### Statistical analyses

First, we explored the bivariate correlations between the five MCQ-30 subscales and the three CANTAB subtests. Any statistically significant correlations (two-tailed tests) were then examined further using hierarchical multiple linear regression analyses, with the MCQ-30 subscale as the dependent variable and the CANTAB subtest as the predictor variable. Bonferroni corrections for multiple comparisons were applied. Control variables were age, education level (ISCED), general cognitive functioning (WAIS PC and SI), and current symptoms of depression (BDI), and anxiety (BAI). Control variables were entered in step 1, and the CANTAB variable in step 2. All statistical analyses were performed in IBM SPSS 22.

## Results

### Sample characteristics

Six participants were excluded from the analyses because of incomplete assessment, and one participant was excluded because of an extremely high score on SWM. A total 299 participants were included in the study. Table 1 presents the demographic and clinical information. There were 201 female participants (67%) and 98 male participants (33%). Fifty-nine participants (20%) were diagnosed with an ongoing episode of major depression, and 54 (18%) had a history of depression. Forty-six participants (16%) had an ongoing anxiety disorder. Fourteen participants (5%) had other mental disorders, and 18 (6%) had personality disorders. Cronbach's alpha for the BDI was 0.96. Computing Cronbach's alpha for the BAI was not possible because only sum scores were available in the data set.

**Table 1:** **Participant characteristics (*N* = 299)**.

**Characteristic**	***M*** **(*SD*)**
Age, years	37.4 (13.1)
ISCED	4.6 (1.0)
BDI	8.8 (11.3)
BAI	5.6 (7.1)
WAIS PC, scaled score	12.6 (3.2)
WAIS SI, scaled score	10.8 (3.0)

*ISCED, International Standard Classification of Education; BDI, Beck's depression inventory II; BAI, Beck's anxiety inventory; WAIS PC, Wechsler Adult Intelligence Scale Picture Completion; WAIS SI, Wechsler Adult Intelligence Scale Similarities*.

### Correlation analyses

Table 2 presents the MCQ-30 subscale scores and CANTAB performance scores. Cronbach's alpha on the MCQ-30 was 0.90 (MCQ-PBW α = 0.79; MCQ-NBW α = 0.83; MCQ-CC α = 0.87; MCQ-NCT α = 0.76; MCQ-CSC α = 0.79). Table 3 presents the bivariate Pearson's correlations between the five MCQ-30 subscales and three CANTAB subtests. Bonferroni correction was applied, indicating that only correlations with significance levels below 0.003 were considered significant at a 0.05 level. Shifting errors correlated positively with negative beliefs about worry [*r*_(297)_ = 0.22, *R*^2^ = 0.05, *p* < 0.001] and beliefs about the need to control thoughts [*r*_(297)_ = 0.21, *R*^2^ = 0.04, *p* < 0.001]. There was a positive correlation between updating errors and negative beliefs about worry [*r*_(297)_ = 0.18, *R*^2^ = 0.03, *p* = 0.002]. There was a positive correlation between updating errors and cognitive confidence at borderline significance level [*r*_(297)_ = 0.17, *R*^2^ = 0.03, *p* = 0.003].

**Table 2:** **Metacognitions and executive control functions**.

**Variable**	***M*** **(*SD*)**
MCQ-PBW	8.5 (2.6)
MCQ-NBW	10.2 (3.9)
MCQ-CC	10.4 (4.2)
MCQ-NCT	9.0 (3.2)
MCQ-CSC	12.4 (4.0)
ID/ED, total errors adjusted	24.8 (21.7)
SWM, between-trial errors	20.0 (17.7)
SST, stop-signal reaction time	189.9 (55.6)

*Twenty MCQ item scores (0.22%) were missing and replaced by the mean of the subscale for each subject. MCQ-PBW, positive beliefs about worry; MCQ-NBW, negative beliefs about worry; MCQ-CC, cognitive confidence; MCQ-NCT, beliefs about the need to control thoughts; MCQ-CSC, cognitive self-consciousness; ID/ED, Intra-extra dimensional task; SWM, Spatial working memory task; SST, Stop-signal task*.

**Table 3:** **Bivariate correlations between metacognitions and executive functions**.

**Variable**	**MCQ-PBW**	**MCQ-NBW**	**MCQ-CC**	**MCQ-NCT**	**MCQ-CSC**
ID/ED	−0.01	0.22^**^	0.10	0.21^**^	0.05
SWM	0.01	0.18^**^	0.17^*^	0.09	0.03
SST	0.03	0.04	0.09	0.09	−0.03

* *p = 0.003*;

** *p < 0.003*.

*MCQ-PBW, positive beliefs about worry; MCQ-NBW, negative beliefs about worry; MCQ-CC, cognitive confidence; MCQ-NCT, beliefs about the need to control thoughts; MCQ-CSC, cognitive self-consciousness; ID/ED, Intra-extra dimensional task; SWM, Spatial working memory task; SST, Stop-signal task*.

### Regression analyses

To control for the possible effect of age, education, general cognitive ability, and current symptoms, we analyzed the statistical significant relationships in four regression analyses. Negative beliefs about worry was predicted by depression and anxiety symptoms (Table 4, step 1). Taking control variables into account, shifting errors predicted negative beliefs about worry (Table 4, step 2). After control variables, updating errors predicted negative beliefs about worry, but the effect was not significant after Bonferroni correction [β = 0.11, *t*_(297)_ = 2.11, *p* = 0.04]. Age, education level, and depression and anxiety symptoms predicted beliefs about the need to control thoughts (Table 5, step 1). After control variables, shifting errors predicted beliefs about the need to control thoughts at a borderline statistical significance level (Table 5, step 2). Cognitive confidence was predicted by age [β = 0.11, *t*_(297)_ = 2.22, *p* < 0.05] and depressive symptoms [β = 0.46, *t*_(297)_ = 5.88, *p* < 0.01], but not by updating errors [β = 0.01, *t*_(297)_ = 0.15, *p* = 0.88].

**Table 4:** **Hierarchical multiple regression with negative beliefs about worry as dependent variable**.

**Predictor**	***B***	***SE***	**β**	***t***	***p***	**95% CI**
**STEP 1 (CONTROL VARIABLES)**						
Age	−0.02	0.01	−0.05	−1.17	0.24	−0.04 to 0.01
ISCED	−0.25	0.19	−0.06	−1.36	0.18	−0.62 to 0.11
WAIS PC	0.00	0.06	0.00	0.08	0.94	−0.11 to 0.11
WAIS SI	0.02	0.06	0.02	0.40	0.69	−0.10 to 0.14
BDI	0.17	0.02	0.50	7.08	<0.01	0.12 to 0.22
BAI	0.12	0.04	0.21	2.95	<0.01	0.04 to 0.19
**STEP 2 (CANTAB VARIABLE)**						
ID/ED	0.03	0.01	0.14	3.15	0.002	0.01 to 0.04

*ISCED, International Standard Classification of Education; WAIS PC, Wechsler Adult Intelligence Scale Picture Completion; WAIS SI, Wechsler Adult Intelligence Scale Similarities; BDI, Beck's depression inventory II; BAI, Beck's anxiety inventory; ID/ED, Intra-extra dimensional task, total errors adjusted*.

**Table 5:** **Hierarchical multiple regression with beliefs about the need to control thoughts as dependent variable**.

**Predictor**	***B***	***SE***	**β**	***t***	***p***	**95% CI**
**STEP 1 (CONTROL VARIABLES)**						
Age	−0.04	0.01	−0.15	−2.70	<0.01	−0.06 to −0.01
ISCED	−0.41	0.18	−0.12	−2.82	0.02	−0.76 to −0.06
WAIS PC	−0.02	0.05	−0.02	−0.37	0.72	−0.13 to 0.09
WAIS SI	−0.04	0.06	−0.04	−0.68	0.50	−0.16 to 0.08
BDI	0.07	0.02	0.24	2.94	<0.01	0.02 to 0.12
BAI	0.10	0.04	0.22	2.57	0.01	0.02 to 0.17
**STEP 2 (CANTAB VARIABLE)**						
ID/ED	0.02	0.01	0.16	3.02	0.003	0.01 to 0.04

*ISCED, International Standard Classification of Education; WAIS PC, Wechsler Adult Intelligence Scale Picture Completion; WAIS SI, Wechsler Adult Intelligence Scale Similarities; BDI, Beck's depression inventory II; BAI, Beck's anxiety inventory; ID/ED, Intra-extra dimensional task, total errors adjusted*.

## Discussion

The present study makes an important first contribution in bridging metacognitions to executive control based on solid cognitive paradigms. We found that negative beliefs about the uncontrollability and danger of worry and beliefs about the need to control thoughts was related to a decreased ability to shift between mental sets. Shifting ability was also associated with metacognitions after controlling for age, education level, general cognitive function, and depression and anxiety symptoms, suggesting that there is a basic association between metacognitions and shifting ability.

Spada et al. (2008b) have emphasized how negative beliefs about the uncontrollability and danger of worry and beliefs about the need to control thoughts create a cognitive gridlock, which produces even more worry. Our results suggest that this cognitive gridlock is associated with decreased ability to shift between mental sets. This may clarify why individuals with high levels of metacognitions experience difficulties with stopping rumination and worry and switching to more adaptive means of coping.

Interpreting the results according to the S-REF model (Matthews and Wells, 2004) could indicate why and how metacognitions are related to shifting ability. Individuals with low executive control fail more often when attempting to stop rumination (Koster et al., 2011). A person who often fails at stopping rumination may conclude that they are not very good at it, which strengthens metacognitions (Fernandez-Duque et al., 2000). Wells and Matthews (1996) refer to this as “belief elaboration.” The association between metacognitions and executive control may be ascribed to this mechanism. However, the association could also be explained by the fact that metacognitions predict rumination, which in turn decreases executive control. Given the cross-sectional design of the study, we cannot draw conclusions on the direction of the relationship.

Reduced inhibitory control has been proposed to be a gateway for negative thought intrusions, and is more likely to trigger rumination (De Raedt and Koster, 2010; Zetsche and Joormann, 2011; Daches and Mor, 2014; Hoorelbeke and Koster, 2017; Koster et al., 2017). Thus, inhibition probably comes into play in the S-REF model when intrusions from low-level networks activate the supervisory executive. There is a close relationship between metacognitions and rumination (Papageorgiou and Wells, 2009; Solem et al., 2016) and between rumination and inhibition (Yang et al., 2016), but metacognitions and intrusions are not directly linked in the S-REF model (Matthews and Wells, 2004). This may explain why metacognitions and inhibition were not related.

Whether low cognitive confidence is an accurate reflection of actual cognitive performance has been uncertain (Wells, 2000). Our results revealed an association between cognitive confidence and updating, but this disappeared after controlling for current depression and anxiety symptoms. This is probably because only currently depressed individuals have lower cognitive confidence (Halvorsen et al., 2015). Thus, the present results suggest that the relationship between low cognitive confidence and executive control is limited and is probably restricted to acute depression and anxiety.

Executive control has been implicated in efficacy of psychotherapy, where symptom reduction after cognitive behavioral therapy (CBT) is primarily produced by its impact on higher-order executive functions (Clark and Beck, 2010). Better executive control predicts the use of adaptive emotion regulation strategies (Schmeichel and Tang, 2015), and the ease with which such strategies are utilized (Gotlib and Joormann, 2010). Compared to CBT, metacognitive therapy (MCT) appears to lead to better improvement of executive control, and it has been speculated that this could be ascribed to the attention training technique (Groves et al., 2015). Attention training in MCT involves listening to different sounds in different spatial locations, then switching attention between these sounds. Attention training may therefore potentially increase shifting ability. However, weakening metacognitions and learning to stop rumination are also important interventions in MCT (Wells, 2011), and appear to focus on increasing shifting ability: stopping rumination involves shifting focus away from one's depressive symptoms, and weakening metacognitions involves switching from rumination as the default mental set of coping to a more flexible mental set (e.g., problem solving). Thus, increased executive control after MCT could also be explained by the weakening of metacognitions.

Individual differences in executive control could be important in the personalization of treatment. Cognitive training which targets shifting ability might increase effectiveness of MCT by increasing the ability to stop rumination and weaken metacognitions. Individuals with low executive control might benefit from training shifting ability before they attempt to stop rumination. On the other hand, individuals with better executive control might not need explicit training. It is also possible that MCT requires a minimum of executive control and that other treatment methods might be more appropriate for individuals with low executive control. Future research should examine how executive control and metacognitions interact during the course of depression and if this is related to treatment.

There are some limitations to the present study, which future research should address. The generalizability of the results is questionable, as the sample was chosen by convenience. Participants recruited via newspaper advertisements and posters may have been influenced by desirability bias when reporting metacognitions. The subtleties of the relationship between metacognitions and executive control could have been elucidated by analyzing the data with individuals separated into sub-groups based on diagnosis, but this was not possible due to low statistical power. After taking into account control variables, the effects of executive control on metacognitions were small to medium in size. Thus, further studies are needed to assess the theoretical and clinical relevance of the current results.

## Author contributions

BK: concept, data analysis and interpretation, drafting and revising the work, and final approval of the version to be published. RJ: data collection. NL: organizing the study. RJ, TS, NL: interpretation of data, critical revision, and final approval of the version to be published. All authors are accountable for all aspects of the work in ensuring that questions related to the accuracy or integrity of any part of the work are appropriately investigated and resolved.

## Funding

The study was funded by The Research Council of Norway (grant number 175387/V50) awarded to NL.

### Conflict of interest statement

The authors declare that the research was conducted in the absence of any commercial or financial relationships that could be construed as a potential conflict of interest.

## Abbreviations

MCQ-PBW: positive beliefs about worry
MCQ-NBW: negative beliefs about worry
MCQ-CC: cognitive confidence
MCQ-NCT: beliefs about the need to control thoughts
MCQ-CSC: cognitive self-consciousness
ID/ED: Intra-extra dimensional task
SWM: Spatial working memory task
SST: Stop-signal task.
